# Adolescent first births in East Africa: disaggregating characteristics, trends and determinants

**DOI:** 10.1186/1742-4755-12-13

**Published:** 2015-02-19

**Authors:** Sarah E Neal, Venkatraman Chandra-Mouli, Doris Chou

**Affiliations:** Senior Research Fellow, Centre for Global Health, Population, Poverty and Policy, Department of Social Statistics and Demography, University of Southampton, Southampton, SO17 1BJ England; Scientist Adolescents and at-risk populations, Department of Reproductive Health and Research, World Health Organization, 20 Avenue Appia,1211, Geneva 27, Switzerland; Medical Officer, Adolescents and At-Risk Populations, Department of Reproductive Health and Research, World Health Organization, 20 Avenue Appia,1211, Geneva 27, Switzerland

**Keywords:** Adolescents, Pregnancy, Low and middle income countries

## Abstract

**Background:**

The use of a single national figure fails to capture the complex patterns and inequalities in early childbearing that occur within countries, as well as the differing contexts in which these pregnancies occur. Further disaggregated data that examine patterns and trends for different groups are needed to enable programmes to be focused on those most at risk. This paper describes a comprehensive analysis of adolescent first births using disaggregated data from Demographic and Household surveys (DHS) for three East African countries: Uganda, Kenya and Tanzania.

**Methods:**

The study initially produces cross-sectional descriptive data on adolescent motherhood by age (under 16, 16–17 and 18–19 years), marital status, wealth, education, state or region, urban/rural residence and religion. Trends for two or more surveys over a period of 18–23 years are then analysed, and again disaggregated by age, wealth, urban/rural residence and marital status to ascertain which groups within the population have benefited most from reductions in adolescent first birth. In order to adjust for confounding factors we also use multinomial logistic regression to analyse the social and economic determinants of adolescent first birth, with outcomes again divided by age.

**Findings:**

In all three countries, a significant proportion of women gave birth before age 16 (7%-12%). Both the bivariate analysis and logistic regression show that adolescent motherhood is strongly associated with poverty and lack of education/literacy, and this relationship is strongest among births within the youngest age group (<16 years). There are also marked differences by region, religion and urban/rural residence. Trends over time show there has been limited progress in reducing adolescent first births overall, with no reductions among the poorest.

**Conclusions:**

Adolescent first births, particularly at the youngest ages, are most common among the poorest and least educated, and progress in reducing rates within this group has not been made over the last few decades. Disaggregating data allows such patterns to be understood, and enables efforts to be better directed where needed.

## Introduction

The importance of the adolescent fertility rate (AFR) as a measure of a nation’s reproductive health is highlighted by its choice as an indicator within the Maternal Health Millennium Development Goal (MDG). Each year, an estimated 16 million girls and young women give birth between the ages of 15 and 19 (around 11% of all births), most of whom live in low or middle income countries [1]. Reliable estimates are available for national adolescent fertility rates (e.g. United Nations Population Division Statistics) which clearly demonstrate very marked differences in trends and levels of adolescent fertility between countries. These national aggregate estimates are valuable in tracking progress at the global level, but fail to provide adequate data to plan projects and programmes or fully evaluate progress within countries. The rate of adolescent fertility is underpinned by complex socio-economic, educational, cultural and service availability factors, and contexts, patterns and trends may differ markedly for different populations within countries. Further, disaggregated data that examines patterns and trends for different groups is valuable in enabling programmes to be targeted at those most at risk. It also enables approaches to be tailored for different populations, depending on the specific determinants or contexts of early pregnancy within specific groups. The proliferation of large-scale, nationally representative surveys such as the Demographic and Household Surveys (DHS) and Multiple Indicator Cluster Surveys (MICS) provide opportunities for many countries to develop much more comprehensive data on adolescent fertility that incorporate some of the contextual, socio-economic and geographic factors. This will enable a much more nuanced and detailed picture of adolescent fertility patterns.

This paper provides detailed analysis on adolescent first births disaggregated by age (<16, 16/17 and 18/19 years)^a^ wealth quintiles, education, region, religion and marital status at birth for Kenya, Tanzania and Uganda. As well as highlighting the importance of including socio-economic, cultural and geographic factors within any comprehensive framework for measuring and monitoring progress in reducing adolescent births, the focus on disaggregated data by age offers a further new dynamic for assessing progress in reducing adolescent fertility: there is some evidence that younger adolescents may face greater risks of poor health outcomes compared to older adolescents, so progress in reducing births in this group is particularly important [2, 3]. In all three countries adolescent pregnancy is identified as both a public health concern and a barrier to development by international and national policy makers, non-governmental organisations and the media e.g. [4–6]. The study examines cross sectional data as well as disaggregated trends over time using DHS studies 18–23 years apart. We also analyse the data using multinomial logistic regression with adolescent first birth at ages <16, 16/17 and 18/19 years as outcomes to examine the determinants of adolescent first birth while adjusting for confounding. The inclusion of three countries allows us to identify similarities in patterns as well as national differences to give some insight into the East African situation as a whole.

## Methodology

For the cross sectional data, the most recent DHS is used for Kenya, Uganda and Tanzania, (2008/9, 2011, and 2010 respectively). Initially, cross tabulations were created to ascertain the percentage of women aged 20–24 at time of survey who had their first birth at age less than 16, 16/17 and 18/19, disaggregated by place of residence, marital status at time of giving birth, region, religion, and wealth quintile. Sample weights were applied as necessary to account for differential chances of selection into the sample. For some of the analyses asset scores and quintiles were recalculated separately for urban and rural populations: this overcomes the problem inherent in joint analyses where assets may have different impact in urban and rural settings, and enables us to examine inequities within urban and rural environments separately. The index was constructed using principal component analysis to assign indicator weights using the same process as used by DHS/Macro [7]. As previous studies suggest that responses may often be heaped on years ending with a 5 or 0, we created histograms for age at first birth by individual year to identify this possible issue within our data: however no heaping was found at ages 15 or 20 years [8, 9].

Trend data for adolescent first birth disaggregated by age, urban/rural residence and wealth are taken from DHS datasets covering a period of 18–23 years. Annual percentage rates of change are calculated. The period used to measure trends by wealth is shorter as asset indices were not introduced in DHS until the mid to late 1990s. We did not disaggregate wealth quintiles by urban/rural residence for trends as relatively small samples sizes made trends somewhat difficult to interpret.

While bivariate approaches are valuable in identifying general patterns of socio-economic inequalities and identifying vulnerable groups, interpretation can be difficult as many of the potential explanatory factors are closely correlated. Multinomial multivariate logistic regression is therefore also carried out to predict first births in age groups under 16, 16/17 and 18/19, with women who did not give birth before aged 20 as the comparison group. In order to ensure consistency the analysis used women aged 20–25 years at the time of the survey in this analysis, but in order to check the robustness of the model the analysis was also run on a larger survey of women aged 20–29. This method enables us to determine whether the determinants of early adolescent first birth (<16 years) are different to those for first birth in later adolescence (16/17 years and 18/19 years). The models include variables for urban/rural residence, region (or in the case of Tanzania, zone, as there were so many regions), religion (Kenya and Uganda only: data was not collected for Tanzania), wealth and literacy level. Due to the fact that some girls who gave birth very early would not have reached the age where secondary school commenced prior to giving birth, further more disaggregated variables for education were not used.

## Results

### Adolescent births disaggregated by age

Table 1 shows adolescent first births disaggregated by age for all three countries. Uganda has the highest proportion of women giving birth before 20 years (57%), followed by Tanzania (56%) and Kenya (47%). In all three countries the highest proportions of first births occur in the 18–19 age group. The lowest proportions occur in the under 16 age group, but these are by no means insignificant: In Uganda 12% of women give birth before their 16th birthday, and the figure is 8% for Kenya and 7% for Tanzania. Confidence intervals (CIs) are quite small for all groups. Very few births occur before 14 years of age (details of breakdown by age under 16 years not shown).

**Table 1:** % **women aged 20**–**24 reporting first birth before aged 20 years**, **disaggregated by age** (**95**% **confidence intervals in parentheses**)

	<16 years	16/ 17 years	18/ 19 years	Total <20 years
**Uganda 2011**	12.3, (10.7-13.9)	20.7, (18.8-22.7)	24.2, (22.1-26.3)	57.3, (54.9-59.7)
Number of cases	200	338	395	933
**Kenya 2008/** **9**	8.4, (7.1-9.7)	17.6, (15.8-19.4)	20.9, (19.0-22.8)	46.9, (44.5-49.2)
Number of cases	145	302	358	805
**Tanzania 2010**	7.2, (6.0-8.4)	21.1, (19.3-22.9)	27.5, (25.4-29.5)	55.8, (53.5-58.1)
Number of cases	137	402	525	1064

### Association between urban and rural residence and adolescent childbearing

Table 2 shows first births disaggregated by age and urban rural residence. In all three countries, the proportion of first births in adolescence is higher in rural than in urban areas for all age groups. However, the differential as expressed by the ratio of urban to rural for the <16 years groups in Tanzania and Uganda are markedly smaller than for the other age groups. Uganda in particular appears to have an unexpectedly large proportion of women giving birth before the age of 16 in urban areas (11%). However, as the data on birth is retrospective we cannot be certain whether they were living in urban or rural areas at the time of the birth or migrated at a later date.

**Table 2:** % **women reporting first birth before aged 20 years**, **disaggregated by age**, **urban/** **rural residence and wealth quintile**

Uganda 2011	<16	16/ 17	18/ 19	<20
Urban	10.8	13	15.1	38.9
Rural	12.9	23.4	27.4	63.7
Ratio urban/rural	0.8	0.6	0.6	0.6
**Kenya 2008/** **9**				
Urban	4.4	10.2	16.3	30.8
Rural	10.2	20.9	23	54
Ratio urban/rural	0.4	0.5	0.7	0.6
**Tanzania 2010**				
Urban	6.2	15.5	18.1	39.8
Rural	7.6	23.9	32.1	63.6
Ratio urban/rural	0.8	0.7	0.5	0.6
**Uganda 2011 Wealth quintiles**	**<16**	**16/** **17**	**18/** **19**	**<20**
Lowest	19.5	28.5	27.8	75.8
Second	15.9	26.6	28.3	70.8
Middle	8.9	23.2	29.1	61.2
Fourth	13.1	17.9	27.7	58.7
Highest	8.3	12.8	15.2	36.3
**Ratio richest to poorest quintile**	0.4	0.5	0.6	0.5
**Kenya 2008/** **9 Wealth quintiles**	**<16**	**16/** **17**	**18/** **19**	**<20**
Lowest	20.2	24.9	28.1	73.1
Second	8.6	21.7	25.1	55.4
Middle	9.5	20.7	23.4	53.6
Fourth	6.2	13.9	22.4	42.5
Highest	3.5	12.6	13.4	29.5
**Ratio richest to poorest quintile**	0.2	0.5	0.5	0.4
**Tanzania 2010 Wealth quintiles**	**<16**	**16/** **17**	**18/** **19**	**<20**
Lowest	11.2	24.5	30.6	66.3
Second	6.3	28.1	33.6	68.0
Middle	7.4	22.9	32.8	63.2
Fourth	10.0	18.7	27.4	56.2
Highest	2.9	14.3	17.2	34.5
**Ratio richest to poorest quintile**	0.3	0.6	0.6	0.5

### Association between wealth and adolescent childbearing

As expected, there is a marked economic gradient for women who have a first birth in adolescence. Table 2 shows adolescent age at first birth disaggregated by age and standard DHS wealth quintile. The gradient is greatest among the <16 years group, suggesting births are particularly concentrated among the poorest: In Kenya for instance, women are around six times more likely to have a first birth under the age of 16 if they are in the poorest quintile compared to the richest, whereas they are just over twice as likely to have a first birth at aged 18/19. In both Kenya and Uganda around one in five women report a first birth before aged 16 in the poorest quintile. In all three countries there is relatively little difference in the proportion of first births among adolescents aged 18/19 for the lower four quintiles, with the only marked drop seen in the richest quintile.

**Figure 1:**
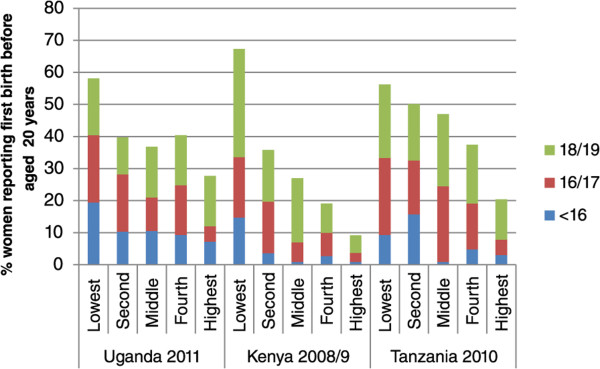
**% women reporting first birth before aged 20 years,** **disaggregated by age and wealth quintile for Uganda,** **Kenya and Tanzania:** **urban only.**

**Figure 2:**
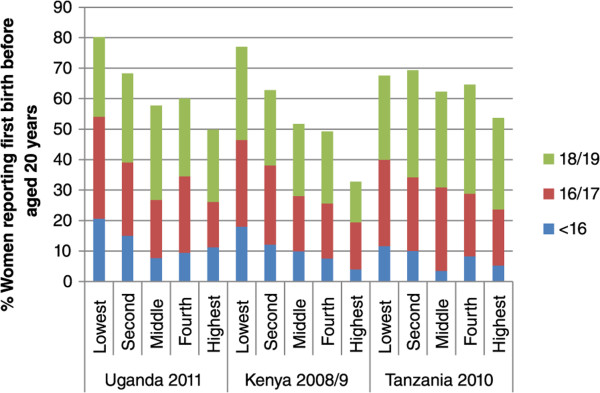
**% women reporting first birth before aged 20 years,** **disaggregated by age and wealth quintile for Uganda,** **Kenya and Tanzania:** **rural only.**

When we present wealth quintiles separately for urban and rural (Figures 1 and 2), we find marked economic gradients for both places of residence. Although small sample sizes mean the findings should be interpreted with some caution, in both urban and rural areas the poorest quintiles have a high proportion of adolescent first births in all age groups. For adolescent first births as a whole the wealth gradient is markedly stronger for urban than rural areas in Tanzania and Kenya in particular, and again is more marked for the younger ages: there is actually no clear gradient for ages 18/19 in Uganda, and rural Tanzania.

### Association between education and adolescent childbearing

In all three countries a very high proportion of women report their first birth in adolescence among those who have had no education or only primary education (Table 3). Again the difference is particularly strong for the <16 age group: for example 27% of women in Kenya who had received no education had given birth before the age of 16 compared with only 3% of those who had received some secondary education. Similar differences can be found in literacy levels, with particularly high levels of adolescent births for younger illiterate adolescents compared to their literate counterparts (Table 3).

**Table 3:** % **women reporting first birth before aged 20 years**, **disaggregated by age and highest education/** **literacy level**

Highest level of education				
**Uganda 2011**	**<16**	**16/** **17**	**18/** **19**	**<20**
No education	20.0	31.3	23.8	75.0
Primary	16.8	25.8	28.6	71.2
Secondary	6.3	15.2	22.2	43.6
Higher	0.8	1.5	3.1	5.4
**Kenya 2008/** **9**	**<16**	**16/** **17**	**18/** **19**	**<20**
No education	26.6	27.4	23.4	77.4
Primary	10.4	23.8	26.9	61.1
Secondary	2.6	7.9	15.0	25.6
Higher	0.0	4.1	2.4	6.5
**Tanzania 2010**	**<16**	**16/** **17**	**18/** **19**	**<20**
No education	13.7	36.2	25.5	75.4
Primary	6.9	21.4	33.1	61.4
Secondary	1.4	4.8	12.3	18.5
Higher	0.0	0.0	0.0	0.0
**Literacy**				
**Uganda 2011**	**<16**	**16/** **17**	**18/** **19**	**<20**
Cannot read at all	21.6	26.5	30.0	78.1
Able to read only parts of sentence	15.0	26.4	29.0	70.4
Able to read whole sentence	7.1	16.4	20.9	44.4
**Kenya 2008/** **9**	**<16**	**16/** **17**	**18/** **19**	**<20**
Cannot read at all	28.8	22.3	21.7	72.8
Able to read only parts of sentence	8.6	29.9	28.9	67.5
Able to read whole sentence	5.5	14.9	19.5	39.9
**Tanzania 2010**	**<16**	**16/** **17**	**18/** **19**	**<20**
Cannot read at all	13.5	31.8	26.8	72.1
Able to read only parts of sentence	14.2	28.3	32.7	75.2
Able to read whole sentence	3.9	16.3	28.2	48.4

### Regional and religious differences in adolescent motherhood

**Figure 3:**
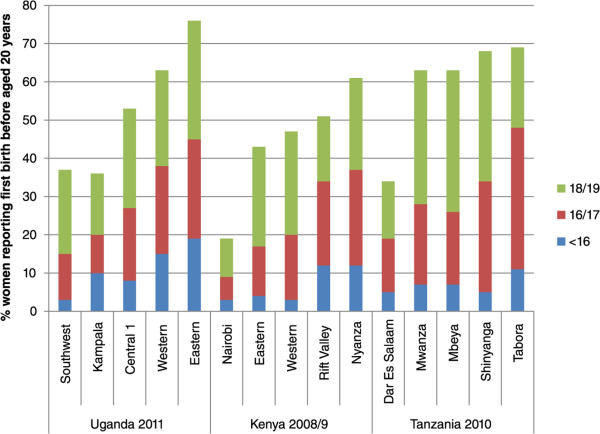
**% women reporting first birth before aged 20 years,** **disaggregated by age and region for Uganda,** **Kenya and Tanzania** **(five regions with largest sample sizes only for each country).**

Figure 3 shows the percentage of adolescent births by age group for the five regions in each country with the largest sample size. Adolescent first births showed marked regional differences across all three countries (particularly Uganda and Kenya) and all three age groups (although small sample sizes mean data disaggregated by age group needs to be viewed cautiously). For instance in Kenya around three times as many women reported a first birth before aged 20 in Nyanza than in Nairobi. The full regions are not presented as for some countries there are many, and sample sizes are very small for some. However, differences may be even greater as many of the regions with smaller populations may be very traditional and rural, with very high rates of adolescent motherhood.

Data on religion are only available for Uganda and Kenya. In Uganda Muslims have a somewhat higher percentage of adolescent first births compared with the Christian religions, and this is most marked in the <16 age group (see Table 4). In Kenya those who state they are Muslim or Catholic have higher percentages of adolescent first births than Protestants, and those who state they are of no religion have markedly higher % of adolescent births.

**Table 4:** % **women reporting first birth before aged 20 years**, **disaggregated by age and religion** (**Uganda and Kenya only**)

Religion				
Uganda 2011	<16	16/ 17	18/ 19	<20
Roman Catholic	12.1	19.2	25.9	57.2
Protestant	9.1	21.9	23.8	54.8
Muslim	16.8	20.4	27.7	65
Pentecostal	13.6	21.5	19.4	54.8
**Kenya 2008/** **9**	**<16**	**16/** **17**	**18/** **19**	**<20**
Roman Catholic	10	16.1	19.2	55.3
Protestant	6.5	17.6	20.8	44.9
Muslim	15.4	13.0	28.5	56.9
Pentecostal	28.9	42.1	15.8	86.8

### Marital status

A higher proportion of women stated they were married at the time of first adolescent birth in Uganda and Tanzania than in Kenya (75%, 72% and 57% respectively - see Table 5). A greater proportion of women were married in rural than in urban areas and the percentage married increased with age for all three groups: for instance in urban Kenya only 30% of women reporting births under 16 years stated they were married, compared to 61% with births aged 18/19 years.

**Table 5:** **proportion of women who report they were married at time of first adolescent birth disaggregated by urban/** **rural residence and age**

	<16	16/ 17	18/ 19	<20
**Uganda 2011**				
Urban	55.6	50.0	73	60.9
rural	69.9	72.8	81	77.8
**Total**	66.7	69.1	80	74.8
**Kenya 2008/** **9**				
Urban	30.4	50.0	61.4	53.0
rural	54.9	57.0	60.7	58.3
**Total**	51.0	55.7	60.9	57.1
**Tanzania 2010**				
Urban	50.0	62.1	59.1	58.8
rural	70.8	70.1	62.1	76.0
**Total**	64.9	68.2	76.9	72.0

### Trends over time in adolescent motherhood

Table 6 shows trends in adolescent first birth by age (with CIs) and Table 7 shows trends by age and urban/rural residence for all three countries. Progress has been very limited during the last two decades in all three age groups, with no consistent differences between the older and younger groups. In Uganda, what little progress has been made is mostly within the younger two age groups, and in Tanzania the only significant change is in the under 16 year group. In Kenya no reduction has been made in the under 16 years group, but significant progress has been made among the 16/17 and 18/19 age groups. When data are further disaggregated by urban/rural residence, we note that in general much more progress has been made in urban areas. The only difference is in the <16 group for Uganda and Tanzania, where there has been little progress or even a slight increase for Tanzania in urban areas, but a marked fall in rural areas. As would be expected with the increased progress among urban women, the differential between the proportion of urban and rural women who have an adolescent first birth has increased in all three countries.

**Table 6:** **Trends over time in** % **women reporting first birth before aged 20 years**, **disaggregated by age** (**CIs in parenthesis**)

Country	Year of first survey		Year of second survey		Average % annual rate of change
Uganda	1988		2011		
**<16**	15.2	(13.0-17.5)	12.3	(10.7 - 13.9)	-0.8
**16/** **17**	26.5	(23.7-29.3)	20.7	(18.8-22.7)	-1.0
**18/** **19**	25.8	(23–28.5)	24.2	(22.1-26.3)	-0.3
**Total** **<20**	67.5	(64.6-70.4)	57.3	(54.9-59.7)	-0.7
**Kenya**	**1988**		**2008/** **9**		
**<16**	8.3	(6.8-9.8)	8.4	(7.1-9.7)	0.1
**16/** **17**	23.7	(21.4-26.0)	17.6	(15.8-19.4)	-1.3
**18/** **19**	26.3	(24–28.8)	20.9	(19.0-22.8)	-1.0
**Total** **<20**	58.4	(55.7-61.1)	46.9	(44.5-49.2)	-1.0
**Tanzania**	**1991/** **2**		**2010**		
**<16**	8.5	(7.2-9.4)	7.2	(6.0-8.4)	-0.8
**16** **/17**	19.8	(18.1-21.7)	21.1	(19.3-22.9)	0.4
**18/** **19**	28.5	(26.5-30.6)	27.5	(25.4-29.5)	-0.2
**Total** **<20**	56.8	(54.6-59.1)	55.8	(53.8-58.1)	-0.1

**Table 7:** **Trends in** % **women reporting first adolescent birth disaggregated by age group and urban**/**rural residence**

	Uganda 1998				Uganda 2011				Annual % rate of change			
	<16	16/ 17	18/ 19	<20	<16	16/ 17	18/ 19	<20	<16	16/ 17	18/ 19	<20
**Urban**	11.5	16.6	23.7	51.8	10.8	13	15.1	38.9	-0.3	-0.9	-1.6	-1.1
**Rural**	15.8	28	26.1	69.9	12.9	23.4	27.4	63.7	-0.8	-0.7	0.2	-0.4
**Ratio urban/** **rural**	0.7	0.6	0.9	0.7	0.8	0.6	0.6	0.6				
	**Kenya 1988**				**Kenya 2007/** **8**				**Annual %** **rate of change**			
	**<16**	**16/** **17**	**18/** **19**	**<20**	**<16**	**16/** **17**	**18/** **19**	**<20**	**<16**	**16/** **17**	**18/** **19**	**<20**
**Urban**	8.3	17.5	24.8	50.6	4.4	10.2	16.3	30.8	-2.3	-2.1	-1.7	-2
**Rural**	8.3	25.7	26.9	60.9	10.2	20.9	23	54	1.1	-0.9	-0.7	-0.6
**Ratio urban/** **rural**	1	0.7	0.9	0.8	0.4	0.5	0.7	0.6				
**Tanzania**	**Tanzania 1992/** **3**				**Tanzania 2010**				**Annual %** **rate of change**			
**Urban 1992/** **3**	5.6	18.9	23	47.5	6.2	15.5	18.1	39.9	0.6	-1.0	-1.1	-0.9
**Rural 1992/** **3**	9.6	20.2	30.6	60.3	7.6	23.9	32.1	63.6	-1.1	1	0.3	0.3
**Ratio urban/** **rural**	0.6	0.9	0.7	0.8	0.8	0.6	0.6	0.6				

Table 8 shows trends by quintiles. In all three countries there is actually an increase in the proportion of women who experience an adolescent first birth for the poorest quintile in all of the age groups (although the increase is very small in Uganda). In Uganda and Tanzania there has also been an increase in the proportion of adolescent first births in the second quintile. By contrast, there are marked reductions in the % of women reporting a first birth under the age of 20 years in the richest quintile for all three countries, and indeed in Tanzania this is the only quintile in which progress has been made. Using Uganda as an example, the proportion of women having a first birth before aged 20 increased by 0.1% for the poorest quintile, and decreased by an average of 2% per annum for the richest quintile. As a result in all countries and in nearly all age-groups the differential (measured as the ratio of births for the richest to poorest) has become greater. It is worth noting that the increase in average % annual rate of change for the poorest quintile is particularly high for the <16 group in Uganda and Kenya.

**Table 8:** **Trends in** % women reporting first adolescent birth disaggregated by age group and wealth quintile*

Uganda	1995				2011				Average annual % rate of change			
	<16	16/ 17	18/ 19	<20	<16	16/ 17	18/ 19	<20	<16	16/ 17	18/ 19	<20
Lowest	17.1	26.6	30.8	74.4	19.5	28.5	27.8	75.8	0.9	0.4	0.0	0.1
Second	20.2	24.3	22.2	66.6	15.9	26.6	28.3	70.8	-1.3	0.6	1.7	0.4
Middle	14.4	27.0	27.3	68.5	8.9	23.2	29.1	61.2	-2.4	-0.9	0.4	-0.7
Fourth	12.0	28.8	30.2	70.8	13.1	17.9	27.7	58.7	0.6	-2.4	-0.5	-1.1
Highest	8.4	18.8	26.3	53.5	8.3	12.8	15.2	36.3	-0.1	-2.0	-2.6	-2.0
Ratio richest/poorest	0.5	0.7	0.9	0.7	0.4	0.4	0.5	0.5				
**Kenya**	**1993**				**2008/** **9**				**Average annual %** **rate of change**			
	**<16**	**16/** **17**	**18/** **19**	**<20**	**<16**	**16/** **17**	**18/** **19**	**<20**	**<16**	**16/** **17**	**18/** **19**	**<20**
Lowest	14.6	24.1	26.3	65.0	20.2	24.9	28.1	73.1	2.4	0.2	0.4	0.8
Second	13.7	21.2	29.4	64.3	8.6	21.7	25.1	55.4	-2.3	0.1	-0.9	-0.9
Middle	6.5	19.4	27.9	53.8	9.5	20.7	23.4	53.6	2.9	0.4	-1.0	0.0
Fourth	7.8	17.9	23.0	48.7	6.2	13.9	22.4	42.5	-1.3	-1.4	-0.2	-0.8
Highest	5.6	13.9	18.1	37.6	3.5	12.6	13.4	29.5	-2.3	-0.6	-1.6	-1.3
Ratio richest/poorest	0.4	0.6	0.7	0.6	0.2	0.5	0.5	0.4				
**Tanzania**	**1996**				**2010**				**Average annual %** **rate of change**			
	**<16**	**16/** **17**	**18/** **19**	**<20**	**<16**	**16/** **17**	**18/** **19**	**<20**	**<16**	**16/** **17**	**18/** **19**	**<20**
Lowest	10.7	27.2	24.5	62.5	11.2	24.5	30.6	66.3	0.3	0.0	0.9	0.4
Second	6.9	17.0	30.0	53.9	6.3	28.1	33.6	68.0	-0.5	8.1	7.0	1.9
Middle	7.1	18.4	30.3	55.8	7.4	22.9	32.8	63.2	0.3	15.9	5.6	0.9
Fourth	7.7	17.2	29.8	54.6	10.0	18.7	27.4	56.2	1.9	10.2	4.2	0.2
Highest	4.5	14.5	22.3	41.3	2.9	14.3	17.2	34.5	-2.2	15.6	1.3	-1.2
Ratio richest/poorest	0.4	0.5	0.9	0.7	0.3	0.6	0.6	0.5				

*Please note baseline year is for a later year than for overall/urban/rural trends as data on wealth quintiles not available for earlier surveys.

### Multivariate analysis of factors associated with adolescent first birth

Tables 9 and 10, 11 show the results of the multivariate multinomial regression which compares women who gave birth before 16 years, 16–17 years and 18–19 years with women who did not give birth below the age of 20 for all three countries. Results are presented as odds ratios (ORs). In all three countries a number of regions have significantly increased ORs, but the patterns vary between the age groups. In Uganda women of the Muslim faith experienced an increased OR of first birth aged <16, 16/17 and 18/19 in comparison to no first birth before aged 20 compared to the reference group (Protestant). In Kenya women have a significantly increased OR of a birth <16 if of the Catholic faith compared to Protestants.

**Table 9:** **Results of multinomial logistic regression for Uganda**: **outcome variables - first birth** <**16 years**, **16/** **17 years and 18/** **19 years** (**comparison group no birth under the age of 20 years**)

	Age <16			Age 16/ 17			Age 18/ 19		
		95% CI for Exp (B)			95% CI for Exp (B)			95% CI for Exp (B)	
	Exp (B)	Lower	Upper	Exp (B)	Lower	Upper	Exp (B)	Lower	Upper
Rural	0.71	.38	1.33	1.12	0.66	1.90	1.57	0.93	2.66
Reference: South western									
Kampala	6.52**	2.17	19.59	2.38*	1.08	5.23	2.52**	1.25	5.07
Central 1	5.79**	2.04	16.39	3.90**	2.05	7.45	2.48**	1.42	4.32
Central 2	9.93**	3.66	27.00	5.14**	2.72	9.71	2.24*	1.24	4.04
East Central	8.34**	3.13	22.24	3.23**	1.68	6.22	1.96*	1.09	3.52
Eastern	9.96**	3.91	25.35	3.91**	2.14	7.13	3.06**	1.82	5.14
Northern	9.55**	3.42	26.65	4.31**	2.14	8.70	2.34*	1.23	4.48
Karamoja	0.68	.13	3.52	2.04	0.81	5.17	1.06	0.43	2.62
West Nile	1.26	.38	4.23	1.15	0.53	2.53	1.08	0.56	2.08
western	9.45**	3.61	24.72	3.51**	1.90	6.49	2.27**	1.34	3.85
Highest quintile (ref)									
Lowest	4.59**	2.10	10.02	4.10**	2.17	7.75	2.88**	1.58	5.27
Second	3.46**	1.66	7.19	3.19**	1.78	5.74	2.66**	1.54	4.60
Middle	1.82	.90	3.71	2.50**	1.47	4.24	2.40**	1.47	3.92
Fourth	3.12**	1.61	6.05	1.90*	1.11	3.28	2.49**	1.54	4.03
Catholic (ref)									
Protestant	0.74	.47	1.17	1.22	0.85	1.74	0.92	0.66	1.28
Muslim	2.23**	1.30	3.82	1.95*	1.21	3.15	1.79*	1.17	2.76
Pentecostal	1.11	.64	1.92	1.21	0.77	1.90	0.77	0.50	1.20
religion - other	2.12	.66	6.81	0.89	0.24	3.33	0.41	0.08	2.00
literacy - whole sentence (ref)									
literacy - cannot read sentence	6.99**	4.47	10.91	2.93**	2.01	4.28	2.79**	1.95	3.98
literacy - part of sentence	4.45**	2.57	7.71	2.49**	1.58	3.90	2.38**	1.56	3.64

*Significant at the 5% level **Significant at the 1% level.

**Table 10:** **Results of multinomial logistic regression for Kenya**: **outcome variables - first birth** <**16 years**, **16/** **17 years and 18/** **19 years** (**comparison group no birth under the age of 20 years**)

	Age <16			Age 16/ 17			Age 18/ 19		
		95% Confidence interval for Exp (B)			95% Confidence interval for Exp (B)			95% Confidence interval for Exp (B)	
	Exp (B)	Lower	Upper	Exp (B)	Lower	Upper	Exp (B)	Lower	Upper
Rural	1.02	0.47	2.23	1.78*	1.06	3.00	0.86	0.54	1.38
Central (ref)									
Nairobi	10.32	0.63	168.25	0.70	0.28	1.72	0.59	0.29	1.21
North Western	17.01	0.94	307.36	2.16	0.54	8.65	0.54	0.15	1.92
Coast	28.81**	2.00	415.00	2.28	1.03	5.02	1.60	0.84	3.06
Eastern	6.46	0.45	92.99	1.27	0.65	2.50	1.18	0.70	1.99
Nyanza	40.51*	3.03	541.70	3.65**	2.01	6.64	1.74*	1.05	2.88
Rift Central	27.19*	2.05	360.93	2.21	1.24	3.95	0.90	0.55	1.46
Western	6.02	0.40	90.21	1.47	0.74	2.91	1.19	0.69	2.06
Protestant									
Catholic	1.77*	1.11	2.83	1.00	0.70	1.42	0.99	0.71	1.36
Muslim	0.96	0.38	2.43	0.68	0.30	1.54	1.31	0.72	2.39
No religion	2.36	0.71	7.85	4.10	1.41	11.89	1.62	0.48	5.51
Highest quintile (ref)									
Lowest	6.24**	2.50	15.56	1.65	0.91	3.00	4.11**	2.28	7.42
Second	2.68*	1.09	6.55	1.11	0.64	1.93	2.44**	1.41	4.23
Middle	3.26*	1.37	7.77	1.15	0.67	1.96	2.33**	1.37	3.97
Fourth	2.01	0.90	4.48	0.78	0.47	1.29	1.76*	1.11	2.79
literacy - whole sentence (ref)									
literacy - cannot read sentence	6.73**	3.78	12.00	2.39	1.40	4.08	1.59	0.96	2.65
literacy - part of sentence	2.11*	1.15	4.00	3.16	2.09	4.77	2.24**	1.49	3.35

*Significant at the 5% level **Significant at the 1% level.

**Table 11:** **Results of multinomial logistic regression for Tanzania**: **outcome variables - first birth** <**16 years**, **16/** **17 years and 18/** **19 years** (**comparison group no birth under the age of 20 years**)

	<16 years			16/ 17 years			18/ 19 years		
		95% Confidence interval for Exp (B)			95% Confidence interval for Exp (B)			95% Confidence interval for Exp (B)	
	Exp (B)	Lower	Upper	Exp (B)	Lower	Upper	Exp (B)	Lower	Upper
rural	.95	.53	1.70	1.57*	1.04	2.37	1.78**	1.23	2.58
Zanzibar (ref)									
Western	3.49	.56	21.71	3.42**	1.38	8.49	2.90*	1.28	6.60
Northern	3.87	.62	24.07	1.63	0.64	4.15	1.80	.78	4.16
Central	4.22	.62	29.01	4.96**	1.87	13.21	4.31**	1.77	10.50
Southern Highlands	2.92	.46	18.65	1.89	0.74	4.84	3.12**	1.37	7.13
Lake	3.49	.56	21.80	2.78*	1.11	6.91	3.47**	1.54	7.83
Eastern	4.61	.74	28.86	2.52*	1.00	6.35	2.53*	1.10	5.83
Southern	5.06	.75	34.08	4.55**	1.72	12.03	5.13**	2.15	12.26
Highest quintile (ref)									
Lowest	3.10**	1.30	7.41	.94	.53	1.65	1.32	.79	2.19
Second	2.53*	1.03	6.21	1.35	.79	2.31	1.61	.99	2.61
Middle	3.10**	1.32	7.28	1.23	.73	2.08	1.55	.97	2.46
Fourth	4.25**	2.11	8.55	1.27	.82	1.98	1.51*	1.01	2.24
Literacy - whole sentence (ref)									
Literacy - cannot read sentence	5.46**	3.51	8.49	3.19**	2.37	4.30	1.49**	1.12	1.99
literacy - part of sentence	6.89**	3.41	13.92	3.47**	2.00	6.05	2.30**	1.35	3.92

*Significant at the 5% level **Significant at the 1% level.

In Uganda the poorer four quartiles are associated with greater odds of birth at ages 16/17 and 18/19 compared with the richest quintile, and the lowest, second and fourth quintile are associated with increased risk for births to the youngest age group (<16). In Kenya the poorest four quintiles and poorest three quintiles have significant ORs for age groups <16 and 18/19 respectively, but no quintiles were significant for the 16/17 age group. In Tanzania all four poorer quintiles are all associated with large and significant increased OR of birth <16, but no quintiles are significant for the 16/17 age group and quintile 4 alone is significant for the 18/19 age group.

When literacy is considered, in Uganda and Tanzania ORs are large and significant for women who cannot read or can only read part of a sentence. There is a clear pattern of higher ORs for the risk of giving birth <16 compared with 16/17 and 18/19. In Kenya the pattern is similar, except the OR is not significantly increased for women who cannot read for births at age 16/17.

Rerunning the analysis using a larger sample of all women aged 20–29 at the time of survey produces very similar patterns (although with generally smaller CIs). The pattern for wealth was clearer for this sample than for the 20–24 age group: in all three countries a greater number of wealth quintiles were associated with increased risk of birth for all age groups, and there was a clear pattern of ORs being larger for the equation with births under aged 16 as an outcome.

In summary, wealth, education and region are all significantly associated with increased odds of an adolescent first birth compared with no adolescent first birth. The same variables are broadly significant when we consider births <16 years, 16/17 years and 18/19 years, but there tends to be a pattern of ORs being greater for the risk of births <16 years.

## Discussion

In all three countries, and particularly in Uganda, a significant proportion of women (7-12%) still report that their first birth was before the age of 16 years. This is concerning, particularly as trends suggest that little progress in reducing births in this youngest group has been made over the last few decades. These girls are likely to be the most vulnerable: a higher proportion are poor, and are more likely to have received little or no education than older adolescent mothers. They are also less likely to be married, which, while not uncommon, in the context of East Africa may carry considerable stigma and lead to social exclusion [10].

It is estimated that up to around 2.5 million births occur to girls aged under 16 years in developing countries each year [11]. However, due to lack of disaggregated data early adolescent childbearing is a largely hidden problem which is rarely recognised or addressed through policy or programmes. Around half of these occur in girls under the age of 15 [11], so are therefore excluded from most official estimates of adolescent fertility as well as maternal mortality estimates. While recent studies suggest that overall adolescents may not experience a greater risk of maternal mortality than women in their 20s [12], there is some limited evidence that younger adolescents (defined as under 15 or under 16 years of age) experience a higher risk of maternal mortality and severe morbidity than older adolescents [3, 13]. There is more robust evidence that birth outcomes for very young adolescent mothers are particularly poor in terms of increased rate of neonatal mortality and stillbirth and lower birth weights [3, 4, 14].

The relatively large number of very early adolescent births, coupled with the potential increased risks faced by both mother and baby strongly points to the need for sexual and reproductive health programmes to be targeted at younger adolescents. However the needs of this group have been largely ignored. Girls in many developing countries are now maturing physically at an earlier age [15] and in Tanzania and Uganda around 15% of women aged 20–24 reported sexual debut before aged 15: in Kenya the figure is slightly lower at 10% [16–18]. Conventional approaches to reducing adolescent pregnancies may be less effective for this group, and there may be a need to develop and tailor interventions to ensure they are appropriate for younger adolescents. Cognitive and emotional differences between younger and older adolescents may require different approaches to address ensure information matches the level of understanding of younger girls [19]. Many will have little or no education, so school-based programmes will fail to reach them, and lack of autonomy may lead to greater transport or monetary barriers in accessing family planning advice or other education initiatives. As younger adolescents may be less focussed on reproductive health issues a more holistic approach that engages them more broadly on their transition to adulthood may be more appropriate [20].

The study suggests a strong relationship between education and literacy, and adolescent motherhood. This has been well recognised in earlier studies [21, 22], but our work highlights that the association is strongest amongst the girls who give birth at the youngest ages. Obviously the relationship between teenage pregnancy and education can operate in two directions: girls with no or limited education may be at more risk of pregnancy, or girls who become pregnant may be forced to curtail their education. The study is not able to prove causality, but it should be noted that women with no education (which obviously has not been curtailed as a result of pregnancy) have very high risks of pregnancy, suggesting education has a protective effect. The fact that ORs for literacy from the multivariate analysis remained large and significant even after adjusting for wealth and region supports the argument improving access to education may have an important impact of reducing adolescent pregnancy. Further studies using longitudinal data would be valuable to explore the relationship. Similar patterns are noted for wealth, and our study finds very high percentages of adolescent first births among the poorest quintiles for both rural and urban residents. It is well recognised that in both developed and developing countries adolescent pregnancy is associated with deprivation [21, 22] and pregnancy in adolescence can both deepen and perpetuate poverty by reducing livelihood opportunities [23].

The very strong ORs in the multivariate analysis for region even after adjusting for urban/rural residence, literacy and wealth are likely to reflect the considerable ethnic diversity within these countries and suggest that there may be strong cultural driver for adolescent fertility. Programmes will need to understand and incorporate the cultural context, and work with local communities and key opinion leaders in order to plan and implement effective and tailored interventions. A review of successful programmes to reduce the risks of pregnancy and other adverse sexual health outcomes highlight the needs for the involvement of both the community and adolescents themselves in developing culturally sensitive interventions [24].

The data on trends suggests modest declines in adolescent first birth for Kenya and Uganda, but not Tanzania. This period coincides with increasing contraceptive use among adolescents aged 15–19 in all three countries [25]. However, rates of use still remain low: the percentage of unmarried sexually active adolescents aged 15–19 who use any modern method of contraception are 23%, 35% and 35% in Kenya, Uganda and Tanzania respectively [16–18]. Rates among married adolescents are lower at 20% in Kenya, 13% in Uganda and 12% in Tanzania. Usage is markedly lower than for older women in both the married and unmarried group. This clearly points to the need to improve access to contraception for young people, and address the barriers that limit their opportunities to obtain appropriate contraceptives and use them correctly. This will require the provision of sexuality education including contraceptive education, making contraceptives more widely available and easily accessible, and making contraceptive service provision adolescent friendly. [26].

Data on trends suggests that the greatest progress has been made amongst the wealthiest: in fact there is some evidence that the percentage of women in the poorest quintile reporting adolescent first births has actually increased. Similarly far greater progress has been made in reducing adolescent first births in urban than in rural areas (although the high levels among the urban poorest should also be noted, and their particular needs acknowledged). This means that over time adolescent mothers are likely to become increasingly concentrated amongst poor, which may create further marginalisation and disadvantage. Targeted programmes are needed to reduce adolescent pregnancies amongst the most vulnerable, yet often large scale programmes fail to acknowledge the socio-economic dimension of adverse reproductive health outcomes, and out of school youth are poorly served [27]. Programmes based on mass media may differentially benefit the wealthier or those living in urban areas who have greater access to these resources [22], and more focus is needed on developing equitable interventions that reach the poorest.

In the three countries between a half and three quarters of women report they are married at the time of their first adolescent birth. Thus a proportion of these pregnancies may be driven by traditional practices and norms, and encouraged and sanctioned by families and the wider community. While this suggests approaches to reducing pregnancies adolescents may require integration with interventions to reduce early marriage it must be acknowledged that within the East African context, sexual activity prior to marriage is common, so marriage may be driven by pregnancy rather than *vice versa*. Indeed in all three countries a significant proportion of women report the time of marriage within nine months prior to their first birth. Also the definition of how marriage or union is defined may differ: the DHS definition is broad, and includes legal and “traditional” marriages, as well as consensual unions with cohabitation. A fuller understanding of both the context and definition of marriage or unions within different sectors of the communities is important in planning interventions, and will require more detailed qualitative research to understand the association between marriage and adolescent births.

It is worth noting that this study has a number of limitations. Cross-sectional studies using retrospective data of this type are unable to establish causation, and in particular it is impossible to establish with any certainty the extent to which the association between adolescent first birth and poor socio-economic indicators is a determinant or a consequence. DHS are generally considered among the best sources of data on adolescent sexual health, but fertility data are known to be affected by recall issues, including possible backdating of births to avoid completing the child module (which is only requested for children born less than five years before the survey date). In addition as adolescent pregnancy may be sensitive in some cultures it may be affected by the social desirability bias that is known to affect data on adolescent sexual activity [28]. Very early motherhood may be particularly stigmatised, resulting in women overstating their age at first birth, or they may be more likely to report they were married at the time of birth.

These statistics do not include pregnancies that do not end in live births. Data are available within DHS for miscarriages, stillbirths and abortions, but there is clear evidence it is unreliable, with significant under-reporting [29, 30]. An estimated 1.4 million adolescents aged 15–19 have unsafe abortions in Africa each year [31], and the burden is significant in all three countries within this study. Restrictive abortion laws, high rates of premarital sex and restrictions in accessing contraception place adolescents at considerable risk in all three countries within this study [32–34]. Further, more detailed analysis would be valuable to understand more about the proportion of adolescent pregnancies that do not end in live births.

This study highlights the need for further research on the context and experience of adolescents who become mothers in order to inform policy and practice. There is particular need for more comprehensive research on particularly vulnerable groups to identify risk factors and develop interventions: these include the poorest (in both urban and rural areas), very young adolescents, and those not in school. Specific focus is needed on the potential increased risks associated with personal or household circumstances, such as those living in single parent families [35], orphaned children and adolescents or young people living outside family settings. In addition further research is needed into the context of adolescent sexual activity and the relationships within which it occurs: studies in Sub-Saharan Africa suggest that a high proportion of women report that their first experience of sexual activity was coerced [36], and an association has also been demonstrated between physical and sexual abuse in childhood or intimate partner violence and adolescent pregnancy in some settings [37, 38]. Research on these issues will require the development of sensitive and effective research quantitative and qualitative methods for collecting data. More analysis of longitudinal data and qualitative studies are also needed to further explore the complex relationship between poverty and poor education and adolescent motherhood.

The development of more nuanced and disaggregated data is of considerable relevance for the post 2015 global agenda, and is particularly relevant in light of the broad focus on reduction of inequities in the Sustainable Development Goals (SDs) [39]. Adolescent motherhood is of direct importance to a number of the proposed goals including those pertaining to poverty, education, health and wellbeing and gender equality, as well as the proposed maternal health target on ending preventable maternal deaths in order to reach a global target of an MMR less than 70 by 2030 [39]. The SDG agenda recognises the need for capacity strengthening in developing countries to improve the quality of data disaggregated by a range of nationally - relevant characteristics in order to adequately monitor and assess progress, and this paper reinforces the importance of this [39].

## Conclusions

This paper highlights the inequity in adolescent early first births in East Africa and the diversity within countries, and stresses the need for more disaggregated data to plan programmes and monitor progress. It also notes a concerning lack of progress in reducing adolescent pregnancies among the poorest and those in rural areas, and points to a need for greater focus on reaching the most vulnerable - very young adolescents and those who are poor or have little or no education – with effective interventions and support.

## Endnotes

^a^It was decided to use a cut-off of <16 as the youngest grouping as there is some indication that this may be the age below which maternal and neonatal outcomes are most adversely affected^5,6^.

## Authors’ information

SN is a British Academy Postdoctoral Fellow within the Department of Social Statistics and Demography at the University of Southampton. VCM and DC work for the World Health Organization.

## Abbreviations

MDG: Millennium Development Goals
AFR: Adolescent fertility rate
DHS: Demographic and Household Survey
MICS: Multiple Indicator Cluster Survey
CIs: Confidence intervals
OR: Odds ratio.
